# Changes in Roughness and Mechanical Properties of Invisalign^®^ Appliances after One- and Two-Weeks Use

**DOI:** 10.3390/ma12152406

**Published:** 2019-07-28

**Authors:** Alexandra K. Papadopoulou, Aurelie Cantele, Georgios Polychronis, Spiros Zinelis, Theodore Eliades

**Affiliations:** 1 Department of Orthodontics, University of Sydney Dental School, Sydney, NSW 2010, Australia; 2 Clinic of Orthodontics and Pediatric Dentistry, Center of Dental Medicine, University of Zurich, 8032 Zurich, Switzerland; 3 Department of Biomaterials, School of Dentistry, National and Kapodistrian University of Athens, 11527 Athens, Greece

**Keywords:** Invisalign^®^, orthodontic appliances, mechanical properties, relaxation, instrumented indentation testing

## Abstract

The aim of this study was to estimate the possible changes of surface roughness and the mechanical properties of Invisalign^®^ appliances over one- and two-week of service. Forty appliances with attachments were retrieved after the end of orthodontic treatment from different patients. Half of them had been used for one week (1W), and the rest for two weeks (2W). Ten unused Invisalign^®^ appliances were used as the control (CON). An equal number of teeth possessing attachments were cut of aligners deriving from all groups (1W, 2W, and CON), and the Sa, Sq, Sz, Sc, and Sv roughness parameters of the internal surface of the aligner attachment area and the opposite lingual side (which was in contact to enamel) were determined by optical profilometry. Then, ten first molars originating from all groups were embedded in acrylic resin, and were ground and polished. Instrumented indentation testing (IIT) was performed in order to determine the Martens hardness (HM), indentation modulus (E_IT_), and relaxation index (R_IT_), according to ISO 14577-2002. The produced data were statistically processed by one- or two-way analysis of variance (ANOVA) and multiple comparison post-hoc tests (a = 0.05). Both the surface roughness and mechanical properties of the retrieved groups (1W and 2W) showed statistically significant differences compared with CON, but without statistically significant differences between each other. The roughness variables of the as-received material were shown to be reduced after intraoral service demonstrating a wear effect. Ageing has a detrimental effect on the surface roughness and mechanical properties of Invisalign^®^ appliances, although this effect is restricted to the first week of clinical usage.

## 1. Introduction

Invisalign^®^ constitutes an aesthetic orthodontic treatment modality that satisfies patient demands [1,2]. The sequential removable aligner fabrication uses a polyurethane-based thermoplastic material [3,4,5], and involves high-precision stereolithography (SLA) and milling technology. The produced series of aligners are capable of introducing tooth movement in small increments, and have been proven to be effective for mild non-skeletal orthodontic malocclusions that do not require extractions [6,7]. The addition of composite resin tooth attachments increases the aligner’s retention, promotes force application, and improves clinical effectiveness, particularly in cases where rotation or intrusion is required [8,9,10].

Irrespective of the orthodontic appliance used, tooth movement is a biological process, which requires light, continuous forces in order to occur without clinical side effects [11,12]. The Invisalign^®^ regimen dictates the intraoral placement of one aligner for either a one- or two-week period, which is then replaced by its sequential successor. The initial force magnitude depends on the material mechanical properties, which affect its stiffness [13], thickness, and the prescribed tooth displacement. With time, force relaxation sets in, which is associated with polyurethane inherent structural instability, resulting in material softening [14] or residual stress relaxation, due to intraoral ageing [4,15]. Regardless of the actual source of the reduction of the force applied with time under steady strain, this may have clinical implications. Although there is a plethora of evidence on the in vitro variation of the force exerted by aligners in artificial environments [5,16,17], there is very limited data on the in vivo-incurred aligner alterations. 

The introduction of attachments to enhance the control of tooth movement results in the wear and surface alteration of the aligner surface in contact with the attachment, which is further accentuated by the removal and re-seating of the appliance. Changes on composite surface morphology have been previously identified [18], with the resin’s composition, and consequently, hardness, having a definitive role in wear resistance.

The aim of this study was to investigate the alteration of the surface roughness and mechanical properties of the aligners exposed to the oral environment under ordinary service conditions in the following two regimes: for one- and two-weeks. The null hypothesis set is that no significant differences will be allocated between the retrieved and unused Invisalign^®^ material.

## 2. Materials and Methods

### 2.1. Materials

Clinically used Invisalign^®^ (Align Technology, San Jose, CA, USA) appliances were obtained from various patients with no history of parafunctional habits. These aligners were collected from several practices after the end of treatment, in an anonymized manner, without patient details, and were only identified as having a one-week and two-week service time. The anonymity of the retrieved specimens precluded the tracking of patient-related data. During treatment, the routine recommendations given to the patients were that they were to remove their devices only for eating and performing oral hygiene. Two major sample groups (1W and 2W) were created according to the aligner intraoral exposure time, which varied from a one- to two-week period. Non-exposed, as-received Invisalign^®^ appliances were used as a control (CON).

### 2.2. Optical Profilometry

An equal number of teeth possessing attachments were cut of aligners deriving from all groups exposed (1W and 2W) as well as the non-exposed (CON). The specimens underwent thorough ultrasonic and non-abrasive chemical cleaning (Coregaextradent, GlaxoSmithKline plc., Brendford, UK), thus removing the accumulated plaque and calculus remnants. The following roughness parameters of the internal surface of the aligner attachment area and the opposite lingual side (which was in contact with the enamel) were determined: arithmetic mean deviation (Sa), root mean square root (Sq), maximum height of the surface (Sz), core void volume showing the volume of the surface (Sc) that could support from 10% to 80% of the bearing ratio, and the surface volume (Sv) showing the volume of the surface that could support from 80% to 100% of the bearing ratio. An optical profilometer (Wyko NT-1100, Tuscon, AZ, USA) with a 20× nominal magnification lens was employed accordingly, and the studied area involved a rectangle of 231 × 303 μm dimensions. The produced data were statistically processed by two-way ANOVA.

### 2.3. Instrumental Indentation Testing (IIT)

Ten lower first molars from different appliances from each group were cut from retrieved aligners deriving from different orthodontic treatment steps. Ten lower first molars originating from the as received aligners were used as the control. The specimens were embedded in acrylic resin (Verso Cit-2, Struers, Ballerup, Denmark), with their occlusal surfaces parallel to the horizontal plane following ultrasonic cleaning. Then, the samples were ground up to 4000 grit SiC paper under water cooling, and were polished with a water-based diamond suspension (NapR1 DiaPro-Struers) of up to 1 μm in a grinding/polishing machine (Dap-V, Struers, Ballerup, Denmark). Instrumented indentation testing (IIT) was performed so as to determine the Martens hardness (HM), indentation modulus (E_IT_), and relaxation index (R_IT_). A universal hardness testing machine (ZHU0.2/Z2.5, Zwick Roell, Ulm, Germany) with a Vickers indenter was employed for that purpose. All of the mechanical properties were measured according to international standard specifications (ISO 14577-2002) [19]. A tetragonal pulse was applied, where the device applied a constant indentation depth of 30 μm for 60 s. The HM was calculated at t1 (where the maximum indentation depth was reached) and R_IT_, according to the following formula:

R_IT_ 30/60 = 100% × (F_t2_ − F_t1_)/F_t1_ (1)

where 30 stands for the indentation depth in μm, 60 for the time holding the constant indentation depth in sec, and F_t1_ and F_t2_ correspond to force values at time t1 and t2, respectively. E_IT_ was measured during the unloading curve, and the Poisson’s ratio of the Invisalign^®^ material was set at ν = 0.43 [4].

### 2.4. Statistical Analysis

The data of the surface parameters and mechanical properties data were initially checked for normality and homoscedasticity by the Kolmogorov–Smirnov and equal variance test, respectively. All of the roughness data were statistically analyzed employing two-way ANOVA with time (one or two weeks) and condition (as-received and retrieved) as discriminating variables, and the Tukey post-hoc multiple comparison test for significance differences among the groups tested. All of the mechanical properties were analyzed by one-way ANOVA and Tukey post-hoc multiple comparison tests, and ANOVA on Ranks and Dunn’s multiple comparison test in the cases of absent of normal distribution or homoscedasticity. For all of the above-mentioned statistical tests, the level of significance was set at 95% (a = 0.05)

## 3. Results

### 3.1. Optical Profilometry

**Figure 1:**
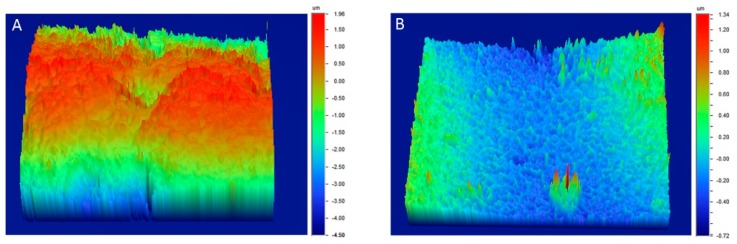
Representative 3D optical profilometric images from the control (CON) (**A**), and one-week (1W) and two-week (2W) groups (**B**). Please note the differences between scales.

**Figure 2:**
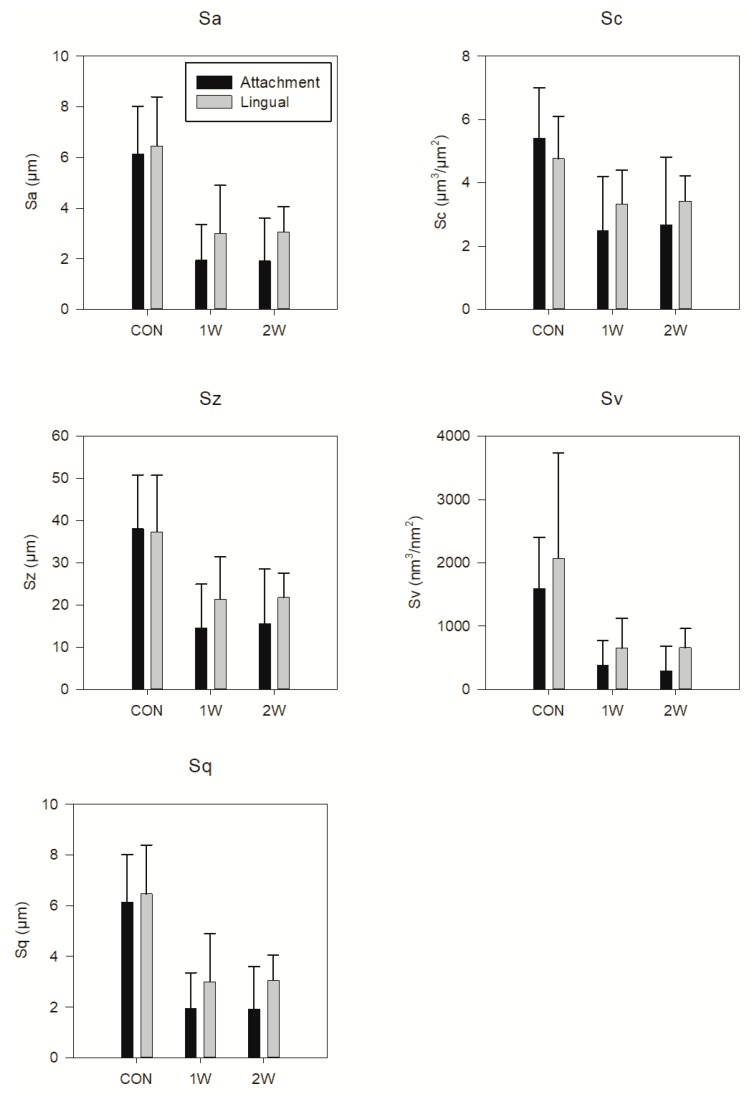
Mean values and standard deviations of all of the roughness parameters tested. Statistical differences are the same for all of the parameters. Statistical differences were significant only between the CON, and 1W and 2W groups for both the lingual and attachment surfaces. Horizontal bars of statistical differences have been omitted for the sake of clarity.

Figure 1 demonstrates a representative 3D reconstruction of the internal surfaces from the attachment and lingual regions of CON (A), and the 1W (B) and 2 W (B) groups. It is evident that the 1W and 2W groups depict a smoother surface compared to CON. The surface roughness parameters variation as a function of the intraoral exposure time and location are demonstrated in Figure 2. The amplitude parameters (Sa, Sq, and Sz) are characterized by a statistically significant reduction of their values from 40% to 70% during first week of clinical usage, whereas the functional ones (Sc and Sv) may even reach up to 80%. The addition of one extra week of intraoral aging does not seem to have an effect on surface roughness. In a similar way, location does not present statistically significant differences.

### 3.2. Instrumental Indentation Testing (IIT)

**Figure 3:**
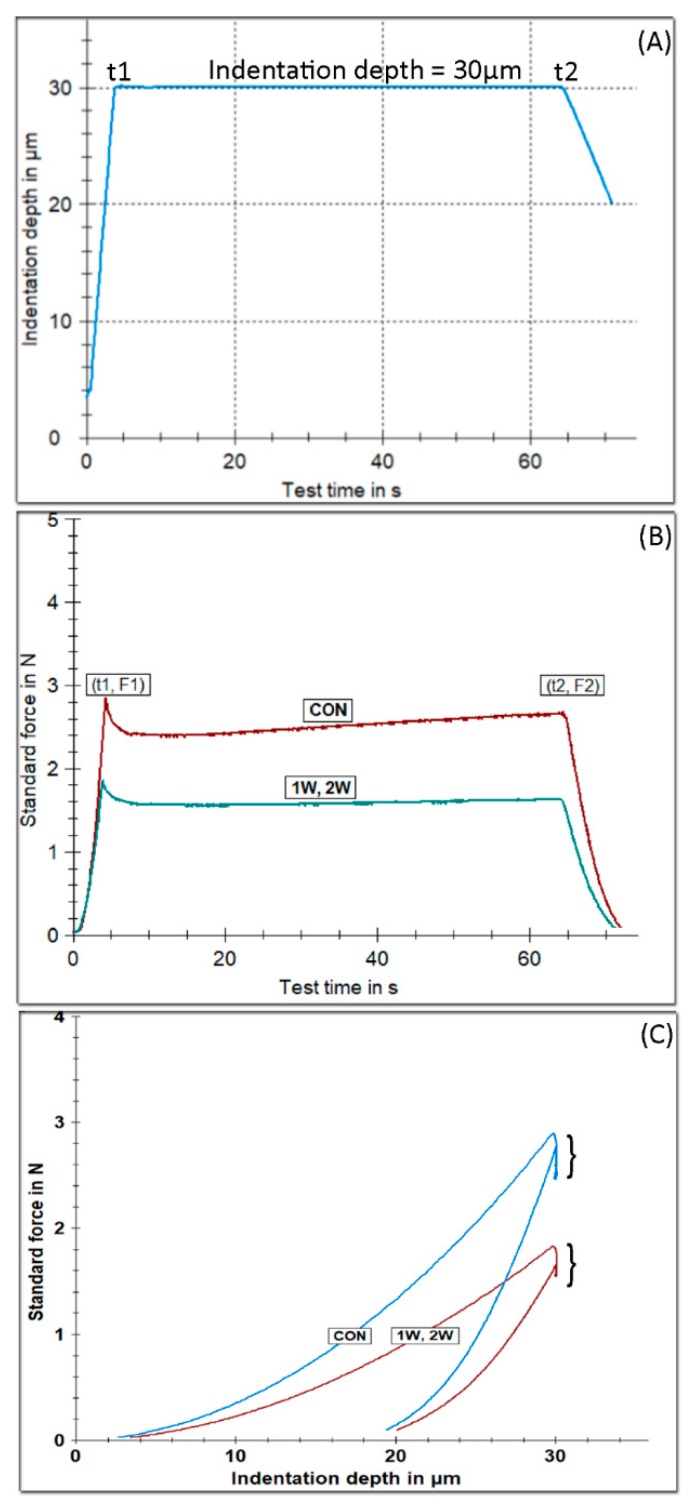
The tetragonal pulse applied for testing the mechanical properties of all of the groups tested, imposing a constant indentation depth for duration of 60 s (**A**). Representative force–time curves showing the force change over the loading period (**B**). Force indention depth curves. The vertical lines pointed by the brackets reflect the time of constant indentation depth (**C**).

**Figure 4:**
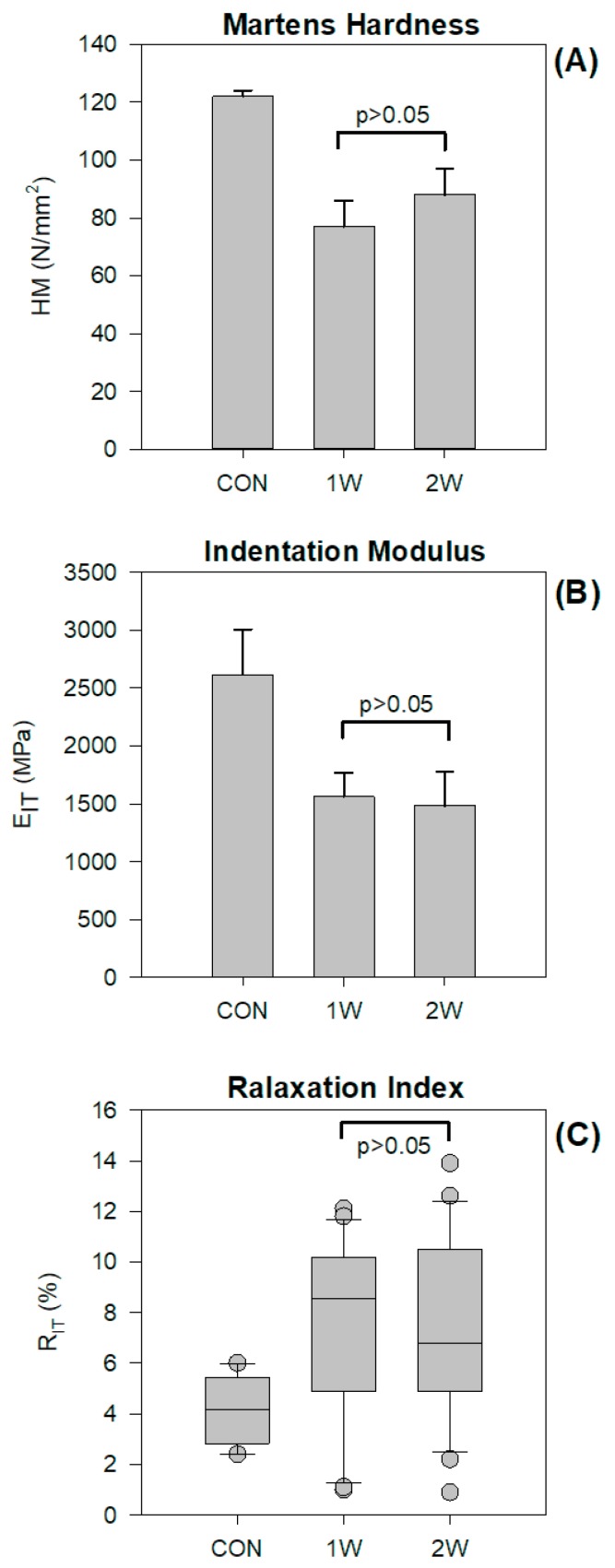
Mean values and standard deviations of the Martens hardness (**A**) and indentation modulus (**B**) of groups tested. Medians, 25% and 75% percentiles, and outliers (demonstrated as circular points) for the relaxation index (**C**). Horizontal lines connect the mean values without statistically significant differences.

Figure 3 illustrates the tetragonal pulse applying a constant indentation depth of 30 μm for 60 s (Figure 3A), representative force over time curves (Figure 3B), and force over indentation depth (Figure 3C), for all of the groups tested. A higher force is required to reach 30 μm of indention depth for the CON group, denoting a harder material compared with the retrieved appliances, as shown in Figure 3B,C. The results of the statistical analysis are presented in Figure 4 for HM (A), E_IT_ (Figure 4B), and R_IT_ 30/60 (Figure 4C). The results for HM and E_IT_ (Figure 4B) showed that the 1W and 2W groups have statistically significant lower values compare with CON. The data for R_IT_ 30/60 are not drawn from normally distributed populations, and thus the median 25% and 75% percentile and outliers are presented in Figure 4C. The statistical analysis demonstrates that 1W and 2W groups have a higher relaxation index, denoting a less resistant material to the relaxation process. No significant differences were identified between the 1W and 2W groups for all of the aforementioned properties.

## 4. Discussion

Based on the results of this study, the null hypothesis must be rejected, as significant differences were found for both the surface roughness parameters and the mechanical properties between the CON and retrieved groups (1W and 2W).

An optical profilometer was used as a non-contact method of 3D surface texture evaluation, because of its high lateral resolution, speed, and reliability. The amplitude surface roughness parameters (Sa, Sq, and Sz) are indicative of the surface roughness of the material, while the functional parameters (Sc and Sv) are associated with the retentive capacity of fluids, and thus plaque accumulation.

The significant reduction of the aligner surface roughness during the first week of orthodontic treatment may lead to a decrease in the material coefficient of friction [20], and thus the micromechanical retention of aligners with resin attachments and enamel. This may, in part, explain the deterioration of the appliance initial retention with time, which is common in clinical praxis, besides the contribution of force attenuation due to the intraoral ageing of the material and tooth displacement.

The non-exposed aligners were characterized by a similar roughness in both surfaces, which can be justified by the reproducibility of the industrial manufacturing process, including stereolithography, milling, and final polishing, without the interference of any human handling. On the other hand, the lower roughness of the 1W and 2W groups should be appended to the intraoral wear of both the aligner attachment and lingual area, with composite and enamel, respectively. The decrease in roughness may also imply a “polishing’” effect from the contact with the much harder enamel or composite resin attachment surfaces. The deformation of appliances during clinical usage may also be implicated in the smoothening of the 1W and 2W groups, although it is anticipated to have a limited effect on the surface roughness.

Commonly used composites for the production of attachment have a hardness in the range of 400 to 600 N/mm^2^ HM [21] (up to six times higher than Invisalign^®^), while human enamel’s hardness has been measured to 2866 N/mm^2^ [22] (almost 23 times higher than Invisalign^®^). Given the huge differences in hardness, it is anticipated that Invisalign^®^ appliances will undergo severe wear by the composite attachment, and also by the enamel in the corresponding lingual surface of the crown during function, and this may explain the surface smoothening of retrieved appliances. Based on the big difference in HM between the composites and enamel, a more intense wear outcome is anticipated for the lingual surfaces, but the surface morphology and roughness may in this case play a more decisive role. In particular, the composite resin attachment area is microscopically characterized by a striation pattern perpendicular to the tooth axis as a positive remnant of the thermoplastic transfer template [18]. In contrast, the tooth enamel surface is lacking this abrasive texture, and appears to be smoother than the non-polished composite resin [23].

Although aligner surface wear may have negative effects on the appliance retentive capacity, this could be proven to be beneficial regarding plaque accumulation and calculus formation [24]. The volume surface roughness parameter, Sv, showed a reduction by almost four times during the first week, which is indicative of a fluid–plaque retention ability reduction. However, the aligner curved internal surface, stagnation of salivary flow, obstruction of tongue, and buccal soft tissue cleaning mechanical action, in combination with a lack of proper oral hygiene, may have detrimental effects on plaque retention and the absorption of various species, leading to the discoloration of Invisalign^®^ appliances. The latter can be commonly seen macroscopically by the calcified internal surface deposit layer formation, especially on the two weeks intraoral exposure positioners.

The rational for selection of IIT for the evaluation of the mechanical properties was twofold, as follows: first, it would had been impossible to manufacture bulky specimens for tensile or bending testing from an Invisalign^®^ appliance, as these are available only in a customized form, and not as a raw material. Therefore, only indentation methods that require limited volume can be applied. In addition, IIT is capable of measuring the additional mechanical properties, such as the modulus of elasticity, relaxation, and creep, according to ISO 14577 [19]. An advantage of IIT methodology over traditional hardness methods (i.e., Vickers Knoop) is that the measurements are independent of the material rebound effect around the indentation, providing values independent of the indentation size effect [25].

Both retrieved groups (1W and 2W) were shown to have inferior mechanical properties compared with CON, denoting that intra-oral ageing has a detrimental effect on the mechanical performance of Invisalign^®^ aligners. The fact that no differences were found between 1W and 2W indicates that the effect of intra-oral aging on the mechanical properties takes place within the first week.

The HM of the CON and retrieved groups (1W and 2W) was found to be similar to the recently reported values obtained by IIT, and the same is true for E_IT_ [4], while, to the best of our knowledge, there is no evidence available on the relaxation of the Invisalign^®^ appliance in vivo, probably because of the requirement of bulky specimens for relaxation testing [26]. The deterioration of mechanical properties has different clinical implications. The decrease in HM denotes a material with decreased wear resistance, and is thus more vulnerable to attrition under occlusal forces. The Invisalign^®^ and other orthodontic appliances are placed in the mouth pre-strained, which leads to the release of orthodontic forces. Under constant deformation, the exerted force is lower (based on the equation σ = Ε*ε, where σ stands for stress, E for elastic modulus, and ε for strain), while the latter indicates that under constant strain, the material is relaxed.

The deterioration of mechanical properties has been assigned to a number of factors. The first is associated with the material itself. Two previous studies demonstrated, through Fourier transform infrared spectroscopy, that Invisalign^®^ is made of a polyurethane-based material [3,4,5], and thus under clinical conditions, suffers from a polyurethane softening mechanism [14]. This mechanism describes that the two-phase microstructure of thermoplastic polyurethane consists of hard and soft segments, where the latter tends to be oriented perpendicular to the applied stresses, and then breaks into smaller pieces in order to receive further deformation. The relief of the residual stresses induced by the manufacturing process and the leaching of matrix plasticizers have also been proposed to explain the degradation of mechanical properties during intra-oral use [4].

From a clinical standpoint, the outcome of this study provides important clinical implications. The deterioration of roughness parameters and mechanical properties has adverse consequences on the retention and orthodontic forces of Invisalign^®^ appliances during orthodontic treatment. The effect of intra-oral aging on the aforementioned properties has been integrated within the first week, and thus, the use of these appliances for two weeks is not recommended.

## 5. Conclusions

Intra-oral aging has a detrimental effect on the surface roughness and mechanical properties of Invisalign^®^ appliances

The deterioration of the aforementioned properties is not time dependent, but has been integrated within the first week of clinical usage.
